# Serial Laboratory Testing for SARS-CoV-2 Infection Among Incarcerated and Detained Persons in a Correctional and Detention Facility — Louisiana, April–May 2020

**DOI:** 10.15585/mmwr.mm6926e2

**Published:** 2020-07-03

**Authors:** Henry Njuguna, Megan Wallace, Sean Simonson, Farrell A. Tobolowsky, Allison E. James, Keith Bordelon, Rena Fukunaga, Jeremy A. W. Gold, Jonathan Wortham, Theresa Sokol, Danielle Haydel, Ha Tran, Kaylee Kim, Kiva A. Fisher, Mariel Marlow, Jacqueline E. Tate, Reena H. Doshi, Kathryn G. Curran

**Affiliations:** ^1^CDC COVID-19 Response Team; ^2^Epidemic Intelligence Service, CDC; ^3^Louisiana Department of Health.

Transmission of SARS-CoV-2, the virus that causes coronavirus disease 2019 (COVID-19), by asymptomatic and presymptomatic persons poses important challenges to controlling spread of the disease, particularly in congregate settings such as correctional and detention facilities (*1*). On March 29, 2020, a staff member in a correctional and detention facility in Louisiana developed symptoms^†^ and later had a positive test result for SARS-CoV-2. During April 2–May 7, two additional cases were detected among staff members, and 36 cases were detected among incarcerated and detained persons at the facility; these persons were removed from dormitories and isolated, and the five dormitories that they had resided in before diagnosis were quarantined. On May 7, CDC and the Louisiana Department of Health initiated an investigation to assess the prevalence of SARS-CoV-2 infection among incarcerated and detained persons residing in quarantined dormitories. Goals of this investigation included evaluating COVID-19 symptoms in this setting and assessing the effectiveness of serial testing to identify additional persons with SARS-CoV-2 infection as part of efforts to mitigate transmission. During May 7–21, testing of 98 incarcerated and detained persons residing in the five quarantined dormitories (A–E) identified an additional 71 cases of SARS-CoV-2 infection; 32 (45%) were among persons who reported no symptoms at the time of testing, including three who were presymptomatic. Eighteen cases (25%) were identified in persons who had received negative test results during previous testing rounds. Serial testing of contacts from shared living quarters identified persons with SARS-CoV-2 infection who would not have been detected by symptom screening alone or by testing at a single time point. Prompt identification and isolation of infected persons is important to reduce further transmission in congregate settings such as correctional and detention facilities and the communities to which persons return when released.

 On March 29, a staff member working in a correctional and detention facility in Louisiana reported symptoms of COVID-19 and later had a positive test result for SARS-CoV-2. Two additional cases among staff members were identified on April 2 and April 10. The facility housed approximately 700 incarcerated and detained persons in 15 dormitories. On April 7, the first case of COVID-19 in an incarcerated person was detected after the patient reported symptoms. During April 8–May 7, through daily active monitoring with temperature screening and oxygen saturation measurements, an additional 35 laboratory-confirmed symptomatic cases were identified among incarcerated and detained persons in five dormitories, resulting in three hospitalizations. Upon identification, all COVID-19 patients were immediately transferred to another facility for medical isolation and care.

During May 7–21, the Louisiana Department of Health and CDC, as part of a public health outbreak response, conducted an investigation to assess the prevalence of infection with SARS-CoV-2 among incarcerated and detained persons residing in quarantined dormitories, and to evaluate symptoms and assess the feasibility of using serial testing with nasopharyngeal swabs to identify additional persons with SARS-CoV-2 infection. Demographic information, medical history, and symptom data were collected for persons in the five affected dormitories using standardized questionnaires. Serial SARS-CoV-2 testing and COVID-19 symptom assessments were conducted on 3 days: May 7 (day 1), May 11, (day 4), and May 21 (day 14). To detect any additional cases before symptom onset, persons who had negative test results on day 1 were retested and completed another symptom assessment on day 4. Those who had negative test results a second time were retested on day 14, the end of the initial quarantine period. On day 14, symptom data were collected again from all persons. The Louisiana Office of Public Health Laboratory tested nasopharyngeal swabs for SARS-CoV-2 using the CDC 2019 reverse transcription–polymerase chain reaction (RT-PCR) panel (*2*), and results were received within 24 hours of testing. Depending on their test results, persons were cohorted by being moved to medical isolation or remaining in quarantined dormitories. Symptom data from all 3 test days were analyzed to classify cases as asymptomatic, presymptomatic, or symptomatic. To identify potential previous illness, outbreak investigators also recorded symptoms reported >2 weeks before testing. Persons were classified as presymptomatic if they reported onset of symptoms after the date of collection of a specimen that had a positive test result; persons were classified as asymptomatic if they had a positive SARS-CoV-2 test result but did not report any symptoms during the previous 2 months or during the 14-day testing period. Attack rates during May 7–21 were stratified by participants’ dormitory assignments on day 1. Analyses were conducted using R (version 3.6.0; The R Foundation). This investigation was determined by CDC to be public health surveillance.^§^ Persons provided voluntary oral consent for testing and questionnaire administration, consistent with the policies of the facility.

At the time of investigation 98 incarcerated and detained persons were in the five quarantined dormitories. All 98 persons were interviewed and tested for SARS-CoV-2 on day 1. The median age was 33 years (interquartile range [IQR] = 29–42 years) (Table 1). The majority of persons tested were male (91, 93%), 65 (66%) were non-Hispanic black, 31 (32%) were non-Hispanic white, one (1%) was non-Hispanic Asian, and one (1%) was Hispanic. Overall, 39 (40%) had an underlying health condition, and 23 (23%) had a body mass index >30 kg/m^2^.

**TABLE 1:** Characteristics of incarcerated and detained persons tested for SARS-CoV-2 in a correctional and detention facility, by dormitory — Louisiana, May 7–21, 2020

Characteristic	Dormitory, no. of residents					
	Dormitory A, 7	Dormitory B, 37	Dormitory C, 11	Dormitory D, 23	Dormitory E, 20	Total, 98
**Age, median (IQR)**	37 (29–47)	31 (29–39)	45 (35–52)	31 (29–36)	37 (29–47)	**33 (29–42)**
**Sex, no. (%)**						
Male	0 (—)	37 (100)	11 (100)	23 (100)	20 (100)	**91 (93)**
Female	7 (100)	0 (—)	0 (—)	0 (—)	0 (—)	**7 (7)**
**Race/Ethnicity, no. (%)**						
White, non-Hispanic	2 (29)	5 (14)	7 (64)	6 (26)	10 (50)	**31 (32)**
Black, non-Hispanic	5 (71)	30 (81)	4 (36)	16 (70)	10 (50)	**65 (66)**
Asian, non-Hispanic	0 (—)	1 (3)	0 (—)	0 (—)	0 (—)	**1 (1)**
Hispanic	0 (—)	1 (3)	0 (—)	0 (—)	0 (—)	**1 (1)**
**Underlying health condition, no. (%)**						
Any	3 (43)	14 (38)	7 (64)	7 (30)	8 (40)	**39 (40)**
Respiratory disease	1 (14)	5 (14)	3 (27)	3 (13)	3 (15)	**15 (15)**
Diabetes	2 (29)	0 (—)	3 (27)	0 (—)	1 (5)	**6 (6)**
Hypertension	2 (29)	7 (19)	5 (45)	3 (13)	3 (15)	**20 (20)**
Other cardiovascular disease	0 (0)	2 (5)	0 (—)	1 (4)	0 (—)	**3 (3)**
Other*	0 (—)	2 (5)	1 (9)	2 (8)	4 (15)	**9 (8)**
**Body mass index >30 kg/m^2^, no. (%)**	2 (29)	7 (19)	1 (9)	7 (30)	6 (30)	**23 (23)**
**Interval from identification of first case to day 1 (May 7), days^†^**	14	20	28	28	30	**—**
**SARS-CoV-2 positive, no. (%)**						
Day 1	4 (57)	20 (54)	6 (55)	10 (43)	13 (65)	**53 (54)**
Day 4	0 (—)	7 (19)	3 (27)	4 (17)	2 (10)	**16 (16)**
Day 14	0 (—)	0 (—)	0 (—)	2 (9)	0 (—)	**2 (2)**
Overall	4 (57)	27 (73)	9 (82)	16 (69)	15 (75)	**71 (72)**

**Abbreviation:** IQR = interquartile range.

* Includes liver disease, immunosuppressive disorder, and neurologic disease.

^†^ Number of days from the identification of the first known COVID-19 case in the dormitory to the first day of serial testing (day 1).

Seventy-one additional cases of SARS-CoV-2 infection were detected in the five dormitories. Among 98 persons tested on day 1, 53 (54%) had positive SARS-CoV-2 test results (Table 2). Among the remaining 45 who had negative test results on day 1, 16 (36%) had positive test results on day 4. Two (7%) of 29 persons who had negative test results on days 1 and 4 had a positive test result on day 14. Of the 71 cases, three (4%) occurred in persons who were presymptomatic at the time of specimen collection, 29 (41%) were in persons who were asymptomatic, and two (3%) were in persons who had had unknown symptom histories. Among the 37 patients who reported COVID-19 symptoms before testing, 11 (30%) reported symptom onset ≤2 weeks before testing, and 19 (51%) experienced symptom onset >2 weeks before testing. Among 27 persons testing negative, 18 (67%) reported COVID-19–compatible symptoms in the previous 2 months, including eight (30%) reporting loss of smell and seven (26%) reporting loss of taste. Among the 98 persons who were tested, 55 (56%) reported at least one COVID-19 symptom during the 2 months before testing, including 37 (52%) who had positive test results and 18 (67%) who had negative test results. Headache (27, 38%) and loss of smell (25, 35%) were the most commonly reported symptoms. During the public health outbreak investigation period, none of the COVID-19 patients identified through serial testing developed severe illness requiring hospitalization.

**TABLE 2:** Reported symptoms* among incarcerated and detained persons (N = 98) in five dormitories in a single correctional and detention facility, by SARS-CoV-2 test results — Louisiana, May 7–21, 2020

**Persons in all five dormitories**	**Subtotal, by symptom, no. (%)**	**Test day, no. tested, no. (%) with positive results**				**All negative, no. (%)**
		**Day 1, 98**	**Day 4, 45**	**Day 14, 29**	**Total all 3 days, 98**	
**Total**	—	**53 (54)**	**16 (36)**	**2 (7)**	**71 (72)**	**27 (27)**
**Symptom status**						
Asymptomatic^†^	37 (39)	19 (36)	9 (56)	1 (50)	29 (41)	8 (30)
Presymptomatic^§^	3 (3)	3 (6)	0 (—)	0 (—)	3 (4)	0 (—)
Symptomatic	55 (56)	29 (55)	7 (43)	1 (50)	37 (52)	18 (67)
Onset in past 2 wks	13 (13)	10 (19)	1 (6)	0 (—)	11 (15)	2 (7)
Onset >2 wks ago	30 (31)	12 (23)	6 (38)	1 (50)	19 (27)	11 (41)
Onset unknown	12 (12)	7 (13)	0 (—)	0 (—)	7 (10)	5 (19)
Specific symptoms*						
Subjective fever	21 (21)	11 (21)	4 (25)	0 (—)	15 (21)	6 (22)
Cough	14 (14)	8 (15)	3 (19)	0 (—)	11 (15)	3 (11)
Shortness of breath	11 (11)	5 (9)	1 (6)	0 (—)	6 (8)	5 (18)
Chills	23 (23)	13 (25)	3 (19)	0 (—)	16 (23)	7 (26)
Muscle aches	24 (24)	15 (28)	3 (19)	1 (50)	19 (27)	5 (19)
Headache	39 (40)	20 (37)	6 (38)	1 (50)	27 (38)	12 (44)
Sore throat	10 (10)	6 (11)	1 (6)	0 (—)	7 (10)	3 (11)
Loss of taste	26 (27)	15 (28)	4 (25)	0 (—)	19 (27)	7 (26)
Loss of smell	33 (34)	20 (38)	5 (31)	0 (—)	25 (35)	8 (30)
Unknown	3 (3)	2 (4)	0 (—)	0 (—)	2 (3)	1 (4)

* During the 2 months preceding the date of data collection.

^†^ During the 2 months preceding testing and during the 14-day testing period. The person who was asymptomatic and had a positive test result on day 14 had not developed symptoms at follow-up 1 week later.

^§^ Persons who reported onset of symptoms after the date of specimen collection, which resulted in a positive test.

The attack rate by dormitory ranged from 57% in dormitory A to 82% in dormitory C. The number of days between the first identified COVID-19 case in each dormitory and day 1 testing ranged from 14–30 days. Dormitory A, which had the lowest attack rate, also had the shortest interval from day of first COVID-19 case to day 1 testing.

## Discussion

High COVID-19 attack rates can occur in correctional and detention facilities (*3*). During May 7–21, among 98 incarcerated and detained persons who were quarantined because of exposure to the virus, 71 (72%) had laboratory-confirmed SARS-CoV-2 infection identified through serial testing. Among those with positive test results, approximately one fourth had positive test results after one or two negative tests at previous time points in quarantine, and 45% did not report any symptoms at the time of testing. These findings suggest ongoing transmission among quarantined persons living in congregate settings; therefore, serial testing of contacts of persons with COVID-19 in correctional and detention facilities can identify asymptomatic and presymptomatic persons who would be missed through symptom screening alone.

Increased detection of SARS-CoV-2 cases by serial testing has been observed in other congregate settings, including homeless shelters and long-term care facilities (*1*,*4*). The high attack rate within these five dormitories and the large proportion of asymptomatic persons with SARS-CoV-2 infection suggest that serial testing of close contacts, including those in congregate settings, should begin immediately after identification of a case to limit further transmission. Some persons infected with SARS-CoV-2 were likely not detected until weeks after they had been infected, which could have contributed to rapid transmission within the quarantined dormitories. Among 71 persons with SARS-CoV-2 identified through serial testing, 27% reported symptom onset 2–8 weeks before testing. Dormitory A, which had the most recent known SARS-CoV-2 introduction among the dormitories, also had the lowest cumulative attack rate, with no additional persons with SARS-CoV-2 infection identified through testing on day 4 or day 14.

CDC’s Interim Guidance on Management of COVID-19 in Correctional and Detention Facilities (*5*), released on March 23, 2020, recommended prompt isolation of COVID-19 patients, quarantine and twice daily symptom-monitoring of exposed persons, and intensified cleaning and disinfection procedures. In these facilities, quarantine is often accomplished by cohorting exposed persons in shared dormitories. It is possible that incarcerated and detained persons experience incentives or disincentives to reporting illness, thereby compromising the effectiveness of symptom screening (*6*,*7*). In addition, asymptomatic or presymptomatic persons infected with SARS-CoV-2 can be missed by symptom screening and transmit the infection (*1*). On June 13, 2020, CDC recommended testing for all close contacts (including those without symptoms) and consideration of broader testing strategies, including the option of widespread and weekly testing of asymptomatic persons, to control transmission in special high-risk settings that have potential for rapid and widespread dissemination of SARS-CoV-2 infection (*8*).

Implementation of symptom screening and serial testing in correctional and detention facilities can be challenging and requires skilled personnel. No single symptom was reported by the majority of persons with COVID-19, and common symptoms such as headache are nonspecific and might not prompt testing. Serial testing is dependent on laboratory capacity and test availability. Delayed receipt of test results inhibits prompt cohorting to reduce transmission from asymptomatic or presymptomatic persons within dormitories. To address these potential challenges, facility staff members should work with their local health department and partners to determine the feasibility of implementing a serial testing strategy. In the future, rapid point of care tests might address some of these challenges.

The findings in this report are subject to at least four limitations. First, serial testing was initiated 2–4 weeks after identification of the first COVID-19 case in an incarcerated person in the dormitories, which likely resulted in substantial transmission before this investigation. Approximately one third of persons with negative test results reported experiencing COVID-19 compatible symptoms >2 weeks before testing and might have already recovered from COVID-19. Second, systematic testing was limited to the five dormitories with known cases among incarcerated and detained persons; staff members were not systematically tested. Exposure to ill staff members might have contributed to transmission. Third, symptom ascertainment might be incomplete, especially for symptoms experienced >2 weeks before testing. Likewise, some persons were unable to provide symptom onset dates. Persons might have underreported symptoms, leading to an overestimate of the prevalence of asymptomatic infection. Finally, this investigation was conducted within five dormitories in one facility; therefore, findings are not generalizable to all correctional and detention facilities.

Approximately 10 million persons are admitted to jails each year, and approximately 55% of detainees return to their community each week.^¶^ Likewise, correctional and detention facility staff members reside in local communities. Thus, high rates of COVID-19 transmission in correctional and detention facilities also have the potential to influence broader community transmission. Because SARS-CoV-2 infection might spread rapidly in correctional and detention facilities (*3*), prevention measures are needed to reduce SARS-CoV-2 introduction and transmission. Mitigation measures should include the quarantine and symptom screening of incarcerated and detained persons upon intake, proper infection prevention and control measures, including the use of appropriate personal protective equipment or cloth face covering** for both staff members and incarcerated and detained persons, regular monitoring of staff members, and encouraging them not to work if they become symptomatic (*5*). Early identification of persons with COVID-19 facilitates their transfer to medical isolation where they can receive timely medical care. Prompt detection and isolation of cases through serial testing might reduce further exposure within the congregate living environment and outside community. Cohorting of incarcerated and detained persons by infection status is essential to slow the transmission of the virus in the facility. Serial testing, particularly in congregate settings is important for identification of persons with SARS-CoV-2 infection and prompt public health response. Reducing transmission in correctional and detention facilities potentially also reduces transmission in communities where staff members live and where detained persons return when released.

**Summary**

**What is already known about this topic?**

Correctional and detention facilities face unique challenges in detecting and mitigating transmission of SARS-CoV-2 infection.

**What is added by this report?**

Testing among quarantined contacts of patients with COVID-19 in a correctional and detention facility identified a high proportion of asymptomatic and presymptomatic cases that were not identified through symptom screening alone. Approximately one fourth of cases were found through serial testing during quarantine.

**What are the implications for public health practice?**

Early detection and isolation of persons with COVID-19, along with testing of close contacts, can slow the transmission of SARS-CoV-2 in correctional and detention facilities. Serial testing, particularly for close contacts of patients, is important for complete identification of cases and prompt public health response in congregate settings.

## Footnotes

* These two authors contributed equally.

† COVID-19–related signs and symptoms include subjective fever, cough, shortness of breath, chills, muscle aches, headache, sore throat, loss of taste, or loss of smell. https://www.cdc.gov/coronavirus/2019-ncov/symptoms-testing/symptoms.html.

§ https://www.ecfr.gov/cgi-bin/retrieveECFR?gp=&SID=83cd09e1c0f5c6937cd9d7513160fc3f&pitd=20180719&n=pt45.1.46&r=PART&ty=HTML.

¶ https://www.bjs.gov/content/pub/pdf/ji16.pdf.

** A person who has trouble breathing, is unconscious, incapacitated, or otherwise unable to remove the mask without assistance should not use a cloth face covering.
